# Comparison of Physiological and Psychological Relaxation Using Measurements of Heart Rate Variability, Prefrontal Cortex Activity, and Subjective Indexes after Completing Tasks with and without Foliage Plants

**DOI:** 10.3390/ijerph14091087

**Published:** 2017-09-20

**Authors:** Sin-Ae Park, Chorong Song, Yun-Ah Oh, Yoshifumi Miyazaki, Ki-Cheol Son

**Affiliations:** 1 Department of Environmental Health Science, Konkuk University, Seoul 05029, Korea; sapark42@konkuk.ac.kr; 2 Center for Environment, Health and Field Sciences, Chiba University, Chiba 277-0882, Japan; crsong1028@chiba-u.jp (C.S.); ymiyazaki@faculty.chiba-u.jp (Y.M.); 3 Department of Horticultural Therapy, Graduate School of Agricultural and Animal Science, Konkuk University, Seoul 05029, Korea; yunahoh@hanmail.net

**Keywords:** brain activity, horticultural therapy, mood state questionnaire, modified semantic differential method, near-infrared spectroscopy, socio-horticulture

## Abstract

The objective of this study was to compare physiological and psychological relaxation by assessing heart rate variability (HRV), prefrontal cortex activity, and subjective indexes while subjects performed a task with and without foliage plants. In a crossover experimental design, 24 university students performed a task transferring pots with and without a foliage plant for 3 min. HRV and oxyhemoglobin (oxy-Hb) concentration in the prefrontal cortex were continuously measured. Immediately thereafter, subjective evaluation of emotions was performed using a modified semantic differential (SD) method and a profile of mood state questionnaire (POMS). Results showed that the natural logarithmic (ln) ratio of low frequency/high frequency, as an estimate of sympathetic nerve activity, was significantly lower while performing the task with foliage plants for the average 3 min measurement interval. Oxy-Hb concentration in the left prefrontal cortex showed a tendency to decrease in the 2–3 min interval in the task with foliage plants compared to the task without plants. Moreover, significant psychological relaxation according to POMS score and SD was demonstrated when the task involved foliage plants. In conclusion, the task involving foliage plants led to more physiological and psychological relaxation compared with the task without foliage plants.

## 1. Introduction 

Sixty-nine percent of humans are expected to live in urban areas by 2050 [1]. Although city dwellers are wealthier and have improved sanitation, nutrition, heath care, etc., urban living is associated with mental health problems such as mood and anxiety disorders [2] and the incidence of schizophrenia [3,4]. Moreover, individuals in modern society have experienced rapid changes such as the emergence of megacities with more than 10 million inhabitants [5] and exposure to more artificial elements. The highly urbanized and artificial environment leads to the exacerbation of human stress levels [1]. Urban upbringing has been shown to affect the perigenual anterior cingulate cortex, which is a key region for regulation of amygdala activity, so that urban living could lead negative affect [6] and stress by increasing amygdala activity [7]. 

Because of these negative influences, attention to human health and well-being from nature is increasing. Experiences with nature can lead to benefits in well-being [8,9,10]. Nature therapy is a health-promotion method, including relaxation by exposure to natural stimuli from forests, urban green spaces, plants, and natural wooden materials, to achieve preventive medical effects [9,11]. 

Previous studies [12,13,14] and a review article [9,10,11,15] have reported the benefits of nature therapy on physiological indicators such as brain, autonomic nervous system, endocrine, and immune activity. Park et al. [16] and Ikei et al. [17] reported that viewing foliage plants led to physiological and psychological relaxation by decreasing prefrontal cortex activity and increasing parasympathetic nervous activity and emotional conditions compared with not viewing foliage plants. Moreover, office workers experienced physiological and psychological relaxation after viewing rose flowers through alterations in parasympathetic nervous activity and emotional conditions [18]. A review article reported that indoor plants provide psychological benefits by reducing stress and increasing pain tolerance [19]. Furthermore, viewing forest scenery increased parasympathetic nervous activity and suppressed sympathetic nervous activity [20,21,22,23,24], and reduced blood pressure and heart rate [21,22,23,25] by enhancing relaxing situations. Igarashi et al. [26,27] and Ikei et al. [28] reported that olfactory stimulation from flower oils, such as rose, orange, perilla essential oil, or Hinoki cypress leaf, led to physiological and psychological relaxation by decreasing oxyhemoglobin (oxy-Hb) concentration in the right prefrontal cortex and increased parasympathetic nervous activity. Moreover, olfactory stimulation with Japanese cedar chips led to a physiological relaxation effect by decreasing total hemoglobin concentration in the left and right prefrontal cortex and systolic blood pressure [29]. Kimura et al. [30] reported exposure to rooms with hiba wood as visual and olfactory stimulation decreased systolic and diastolic blood pressure and salivary amylase activity compared to a room with no hiba wood. Furthermore, a study reported that touching a cypress tree increased parasympathetic nerve activation more than touching marble [31], although research-based evidence for touch stimulation remains lacking.

Meanwhile, actively participating in a transplanting activity with real potted flowers led to a better emotional state compared with transplanting activities using artificial potted flowers [32]. Moreover, a transplanting activity with foliage plant was shown to reduce physiological and psychological stress compared to performing a computer task [33]. However, there is a severe lack of research investigating the physiological responses to activities involving plants as an active participation in nature. The previous studies have measured a physiological index such as heart rate variability (HRV) or prefrontal cortex activity, to verify the effects. 

Therefore, this study aimed to investigate both physiological and psychological responses in transplanting tasks with and without foliage plants as an active participation by measuring HRV, prefrontal cortex activity, and emotional responses.

## 2. Materials and Methods 

### 2.1. Subjects

A total of 24 Korean males in their third decade of life (i.e., 20 to 29 years of age) participated in this study (Table 1). Participants were recruited using a volunteer list provided by Konkuk University (Seoul, Korea), or face to face on the Konkuk University campus. Inclusion criteria were right hand dominance and not having rhinitis symptoms that could affect the collection of physiological data. All subjects provided informed written consent to participate. 

Before starting the experiment, body weight (without shoes) and height of the subjects were measured using a body fat analyzer (ioi 353; Jawon Medical, Gyeongsan, Korea) and an anthropometer (OK7979; Samhwa, Seoul, Korea). Body mass index was calculated for each participant (body mass index = weight [kg]/height [m]^2^). The mean age of the subjects was 23.8 ± 2.6 years. The mean body mass index was in the normal range (22.9 ± 2.8 kg/m^2^ [34]). 

Subjects were required to abstain from caffeine and smoking for at least 2 h before participation in the experiment. Subjects received $20 as an incentive to complete the experiment. The study was approved by the Institutional Review Board of Konkuk University (7001355-201508-HR-079) and the Ethics Committee of the Center for Environment, Health and Field Sciences, Chiba University, Japan (project identification code number: 5). 

### 2.2. Experimental Protocol

**Figure 1:**
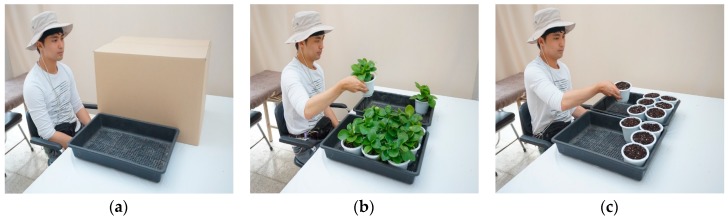
Experimental conditions (**a**) Preparation; (**b**) Task with foliage plants; (**c**) Task without foliage plants.

This study used a crossover experimental design, in which each experimental group crossed over from one treatment to another [35]. Figure 1a presents the resting position before each treatment. The treatment involved a task: transferring pots with foliage plants to a tray (Figure 1b); alternatively, the task after crossover was to transfer pots without foliage plants to a tray (Figure 1c). The foliage plants used for the transfer task were *Peperomia obtusifolia.* The plants have a general green color without any color variation on the leaves. The total weight of each pot, with or without a foliage plant, was approximately 500 g. 

**Figure 2:**
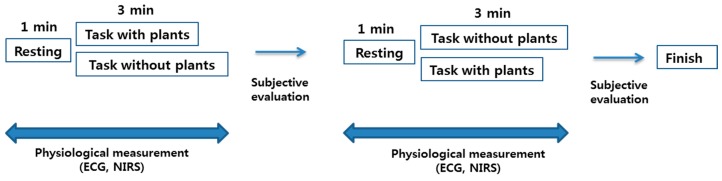
Crossover experimental design protocol for this study. ECG, electrocardiography; NIRS, near-infrared spectroscopy.

Each subject repeatedly performed the task with foliage plants for 3 min and then performed the same task without foliage plants for 3 min (Figure 2). Each subject transferred approximately 33 pots over a 3 min period with their self-controlled speed. The subjects randomly conducted the tasks with or without foliage plants. During the task, physiological parameters, including HRV and oxy-Hb concentration in the prefrontal cortex, were measured using portable electrocardiograph (ECG) and near-infrared spectroscopy (NIRS) devices, respectively. 

Immediately after completing the tasks, subjects subjectively evaluated their emotional state using two different surveys, which took approximately 3 min to complete (Figure 2). 

The experimental environment (1.5 m × 1.7 m) was previously prepared at the Konkuk University campus. The environment was blocked by a curtain to shield from noise or interruption. The temperature, relative humidity, and illumination were 25.4 ± 1.5 °C (mean ± standard deviation), 52.5 ± 5.8%, and 1645 ± 189 lx, respectively. 

### 2.3. Measurement of Heart Rate Variability and Prefrontal Cortex Activity 

To assess autonomic nervous system activity, HRV was measured using a portable ECG (Activtracer AC-301A, GMS, Tokyo, Japan) during the task with and without foliage plants. The ratio of low-frequency (LF) to high-frequency (HF) (LF/HF) components is a measure of sympathetic nervous system activity as one parameter of HRV [36,37]. Frequency spectra were generated using HRV software (MemCalc/Win; GMS, Tokyo, Japan). LF (0.04–0.15 Hz) and HF (0.15–0.40 Hz) were calculated. To normalize HRV parameters, natural logarithmic (i.e., ln) transformed values were used [38].

Each subject wore a portable NIRS device (Pocket NIRS Duo; Dynasense, Shizuoka, Japan) placed on the left and right sides of the forehead [39]. Oxy-Hb concentration in the right and left prefrontal cortex was continuously measured using the portable NIRS device at 1 Hz during the task with and without foliage plants. NIRS is a noninvasive method of monitoring cerebral blood oxygenation, which is not subject to head motion due to temporal resolution of the device [40,41]. Near-infrared light penetrates tissue several centimeters and enables the continuous measurement of hemodynamic parameters in the prefrontal cortex of the brain [41,42]. 

Baseline data for HRV and oxy-Hb concentration in the prefrontal cortex were collected using the portable ECG and NIRS, respectively, while the subjects sat quietly on a chair for 1 min before starting the experiment. 

### 2.4. Subjective Evaluation

To evaluate subjective emotional condition of the subjects for tasks with and without foliage plants, Korean versions of a modified semantic differential (SD) method [43] and a profile of mood state (POMS) [44] questionnaire were used. The subjects answered questions immediately after completing the task with and without foliage plants, which took an average of 3 min (Figure 2).

The modified SD method is a questionnaire consisting of three categories: “comfortable to uncomfortable”; “natural to artificial”; and “relaxed to awakening”. The score for each question is classified according to 13 scales according to the degree of emotion. Subjects answered each question, with a higher score representing better emotional condition [45].

The POMS questionnaire consists of a total of 30 questions, responses to which are scored on a 5-point Likert scale [44,46]. The questions are categorized into six subcategories including tension-anxiety (T-A), depression-dejection (D), anger-hostility (A-H), fatigue (F), confusion (C), and vigor (V). For T-A, D, A-H, F, and C, a lower score represents a better emotional condition. A higher score for V indicates better emotional condition. The total mood disturbance (TMD) score was calculated using the following formula:

TMD score = (T-A) + (D) + (A-H) + (F) + (C) – (V).

A lower total TMD score reflects a better emotional condition [47]. 

### 2.5. Data Analysis

To compare treatment according to HRV and oxy-Hb concentration, a paired *t* test with Holm correction at *p* < 0.05 was performed using SPSS version 20 (SPSS Inc., Chicago, IL, USA). The modified SD method and POMS data were analyzed using the Wilcoxon signed-rank test and the SPSS software. In both cases, one-sided tests were used because the hypothesis was that humans would be relaxed by foliage plants.

## 3. Results and Discussion

### 3.1. Physiological Relaxation

#### 3.1.1. Heart Rate Variability

**Figure 3:**
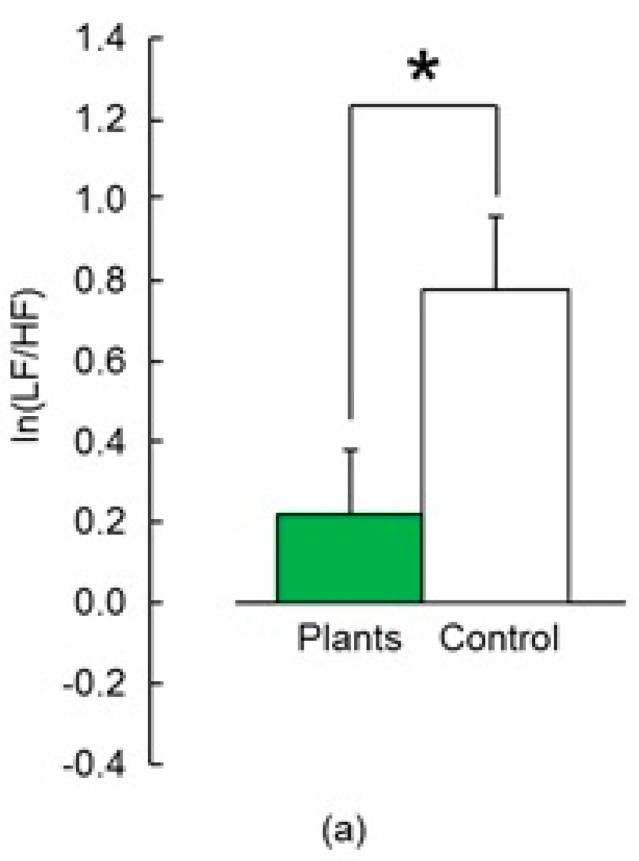

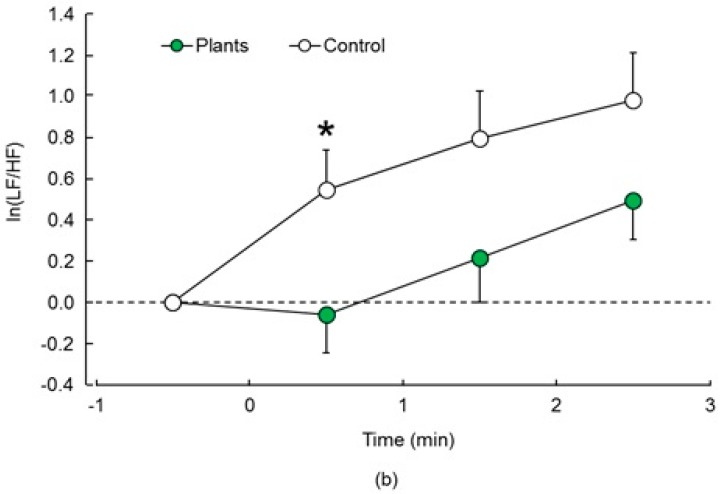
Comparison of ln(low frequency (LF)/high frequency(HF)). (**a**) Mean over 3 min interval. Data presented as mean ± standard error (*N* = 21). * *p* < 0.05 as determined by the paired *t*-test; (**b**) Changes in ln(LF/HF) over time under the two treatment conditions. Data presented as mean ± standard error (*N* = 21). * *p* < 0.05 as determined by the paired *t*-test; Holm’s correction was used.

Values of ln(LF/HF), which estimate sympathetic nerve activity, were significantly lower in the task performed with foliage plants compared to the task without foliage plants (Figure 3a) at *p* < 0.05 by paired *t* test. In the time-series data, a significant difference between the two treatments emerged after the first minute (Figure 3b). Meanwhile, there was significant difference in the 0–1 min (*p* = 0.010, *p* < 0.05 by Holm correction), 1–2 min (*p* = 0.040, *p* > 0.05 by Holm correction), and 2–3 min (*p* = 0.047, *p* > 0.05 by Holm correction) interval. 

In addition, there was no difference for respiration interval between the subjects in the control (0.34 Hz, 20.5 times per min) and experiment (0.33 Hz, 20 times per min) groups by paired *t* test at *p* = 0.47. 

This suggests that the task performed with foliage plants resulted in better physiological relaxation compared with the task without foliage plants. However, future studies are needed to investigate the lasting effects of HRV.

In a previous study, LF/HF ratios were increased with mental tasks compared with resting; however, the differences were not statistically significant [48]. Lee et al. [32] reported that the sympathetic nervous response―ln(LF/[LF+HF])―was significantly lower in subjects performing the transplanting activity with real flowers than with artificial flowers. Another study showed that ln(LF/[LF+HF]) was significantly lower in transplanting work with foliage plants than working with a computer [33]. This suggests that working with real plants results in more physiological and psychological relaxation compared with artificial plants or inanimate objects. Moreover, visual stimulation using real flowers demonstrated lower values for LF/HF in high school students than with artificial flowers [49]. Thus, the present study showed similar results to the previous studies that viewing plants or working with plants decreased sympathetic nervous activity so that it led to physiological and psychological relaxation. 

#### 3.1.2. Prefrontal Cortex Activity

**Figure 4:**
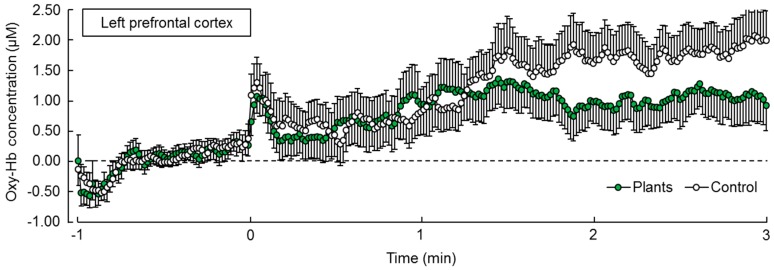
Changes in oxy-hemoglobin (Oxy-Hb) concentration in the left prefrontal cortex over time under the two treatment conditions. Data presented a mean ± standard error (*N* = 21).

Oxy-Hb concentration in the left prefrontal cortex showed a decreased tendency in the task with foliage plants compared to without plants in the 2–3 min interval, although there was no statistically significant difference in the measured data for 3 min at *p* < 0.05 by paired *t* test (Figure 4). Meanwhile, oxy-Hb concentration in the left prefrontal cortex in the 2–3 min interval tended to decrease (*p* = 0.030, *p* < 0.10 by Holm correction). However, there was no statistically significant difference in the 0–1 min (*p* = 0.202) and 1–2 min (*p* = 0.415) intervals of the oxy-Hb concentration in the left prefrontal cortex. On the other hand, oxy-Hb concentration in the right prefrontal cortex was not changed during either task. 

Although only a few similar studies have been conducted, one previous investigation reported results similar to this study. Oxy-Hb concentration in the left prefrontal cortex was lowered more by touching a cypress tree for 90 s than touching marble for 90 s, and led to better physiological relaxation [31]. However, oxy-Hb concentration in the right prefrontal cortex remained unchanged by touch stimulation in both conditions. Previous studies have shown that visual or olfactory stimulation led to a physiological relaxation reflected by oxy-Hb concentration variations in the right prefrontal cortex without changes in the left prefrontal cortex [16,26,27,28]. 

In general, the left hemisphere controls dexterity in the right side of the body, language, the ability to classify, and typical behavior. The right hemisphere specializes in reacting to emergencies, spatial organization, recognizing faces, and processing emotions [50]. The subjects used their right hand because it is possible to activate the left hemisphere; however, the mechanism is unknown. Future study is needed to verify differences in the activation of the left and right hemisphere with left-handed subjects.

### 3.2. Subjective Evaluation

**Figure 5:**
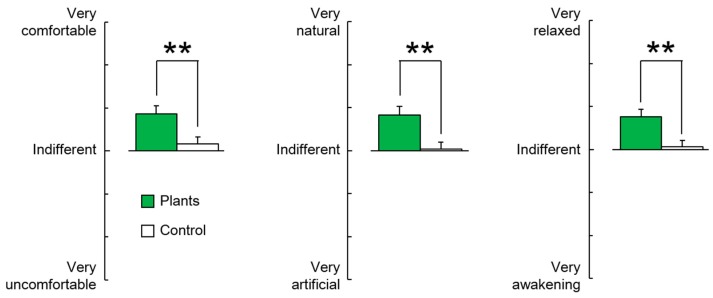
Comparisons of a modified semantic differential (SD) method under conditions of a task with foliage plants versus a task with no foliage plants. Data presented as mean ± standard error (*N* = 24). ** *p* < 0.01 as determined by the Wilcoxon signed-rank test.

Subjective evaluation of emotional condition for the task with or without foliage plants was performed using the SD method and the POMS questionnaire. When the subjects were performing the task with foliage plants, they felt significant positive emotions, such as being more comfortable, natural, and relaxed, compared with the task without foliage plants (Figure 5). 

**Figure 6:**
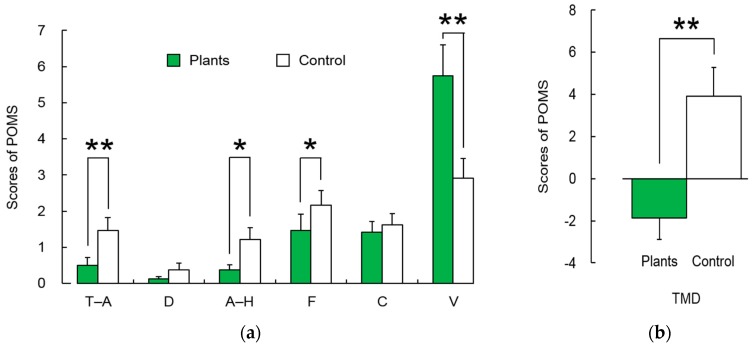
(**a**) Comparisons of tension-anxiety (T-A), depression-dejection (D), anger-hostility (A-H), fatigue (F), confusion (C), and vigor (V) in the profile of mood state (POMS) questionnaire between tasks involving foliage plants and tasks with no foliage plants; (**b**) Comparisons of the total mood disturbance (TMD) score in the profile of mood state (POMS) questionnaire between conditions. Data presented as mean ± standard error (*N* = 24). ** *p* < 0.01; * *p* < 0.05 as determined by the Wilcoxon signed-rank test.

Moreover, subjects reported more positive psychological relaxation in the task involving foliage plants compared with the task without foliage plants (Figure 6a,b). Subcategories such as T-A, A-H, and F were significantly lower, and V was significantly higher when performing the task with foliage plants than without foliage plants (Figure 6a). The TMD score in the task with foliage plants was significantly lower than those without foliage plants (Figure 6b), which suggests that tasks involving foliage plants resulted in a better emotional condition than those without foliage plants. 

Similar findings have been reported in previous studies. Psychological conditions of subjects were better in settings where they were working with or viewing real plants compared to artificial plants [32,33,49]. Subjects who performed a transplanting activity with real flowers reported a better emotional condition than transplanting with artificial flowers [32]. Moreover, when subjects had real flowers as visual stimulation, they exhibited better emotional conditions than viewing artificial flowers [49]. 

The human body is best adapted to natural environments [9,11,51] because early humans devoted more than 99.99% of their time to living in a natural environment. By connecting to nature and working with plants, subjects can release a state of stress caused by highly urbanized and artificial environments [9,11]. Various hypotheses such as Biophilia Hypothesis [14], Kaplan’s attention restoration hypothesis [13], and Ulrich’s stress reduction hypothesis [12] support the benefits from being in or viewing forests, plants, flowers, urban green spaces, parks, and natural wooden materials [52,53] because humans have instinctive longing to nature.

Meanwhile, horticultural activities have been used as a treatment modality in horticultural therapy, which is a complementary alternative medicine. Various horticultural activities with living plants, including digging, raking, planting, weeding, harvesting, and watering, have been defined as healthy physical activities reflecting low- to high-intensity physical activities in children, adults in their 20s, and the elderly (older than 65 years of age) [54,55,56,57,58,59,60]. Moreover, upper and lower limb muscles are actively being engaged while subjects perform various horticultural activities [57,58]. A previous study reported that horticultural activities, such as transferring pots or trays, filling pots with soil, and watering, among others, have characteristics similar to reaching and grasping rehabilitation training in kinematic and kinetic analyses [61]. Therefore, horticultural activities can be used as an adjunct treatment during physical rehabilitation. Results of this study support that working with living plants leads to more physiological and psychological relaxation than working without living plants. 

Stroke patients with depression exhibited significant improvement in depressive symptoms after participating in an eight-session horticultural therapy program [62]. Moreover, elderly women >70 years of age maintained psychological health and improved their cognitive abilities, physical functional abilities, and immune systems by participating in a 15-session gardening intervention [59,63]. Accordingly, horticultural activity intervention has potential as a physical rehabilitation treatment, with the added benefit of physiological and psychological relaxation. In addition, horticultural activity interventions provide goal- and task-oriented tasks using living plants so participants can maintain their motivation for therapy sessions [64] with enjoyment [60,65]. 

## 4. Conclusions

The present study provided research-based scientific evidence supporting the physiological and psychological benefits of tasks involving living foliage plants. Compared to the task without foliage plants, the task involving foliage plants resulted in better physiological and psychological relaxation. ln(LF/HF) values were significantly lower in the task involving foliage plants than in the task without foliage plants. Oxy-Hb concentration in the left prefrontal cortex demonstrated a tendency to decrease in the task involving foliage plants. Moreover, subjective emotional conditions were better in the task involving foliage plants than the task without foliage plants. Our findings suggest that working with foliage plants induces more physiological and psychological relaxation than working without foliage plants. It would be interesting to measure the lasting effects starting time of HRV and prefrontal cortex activity. Future studies should compare the therapeutic effects of horticultural activity intervention and traditional rehabilitation intervention for subjects with impairments and disabilities. Moreover, it is necessary to measure the differences in physiological and psychological responses according to the different senses such as touch, sight, smell, or hearing.

## Figures and Tables

**Table 1:** Descriptive information of subjects who participated in the study (*N* = 24).

Variable	Mean	Std. Dev.
Age (years)	24.2	2.7
Height (cm)	171.1	6.3
Body weight (kg)	67.8	11.4
Body mass index (kg∙m^−2^) ^1^	23.0	2.8

^1^ Body mass index = [weight (kg)/[height (m)^2^].

## References

[1] Dye C. (2008). Health and urban living. Science.

[2] Peen J., Schoevers R.A., Beekman A.T., Dekker J. (2010). The current status of urbanrural differences in psychiatric disorders. Acta Psychiatr. Scand..

[3] Krabbendam L., Van Os J. (2005). Schizophrenia and Urbanicity: A Major Environmental influence—Conditional on Genetic Risk. Schizophr. Bull..

[4] Pedersen C.B., Mortensen P.B. (2001). Evidence of a Dose-Response Relationship between Urbanicity during Upbringing and Schizophrenia Risk. Arch. Gen. Psychiatry.

[5] Kennedy C., Stewart I.D., Ibrahim N., Facchini A., Mele R. (2014). Developing a Multi-Layered Indicator Set for Urban Metabolism Studies in Megacities. Ecol. Ind..

[6] Pezawas L., Meyer-Lindenberg A., Drabant E.M., Verchinski B.A., Munoz K.E., Kolachana B.S., Egan M.F., Mattay V.S., Hariri A.R., Weinberger D.R. (2005). 5-HTTLPR Polymorphism Impacts Human Cingulate-Amygdala Interactions: A Genetic Susceptibility Mechanism for Depression. Nat. Neurosci..

[7] Diorio D., Viau V., Meaney M.J. (1993). The Role of the Medial Prefrontal Cortex (Cingulate Gyrus) in the Regulation of Hypothalamic-Pituitary-Adrenal Responses to Stress. J. Neurosci..

[8] Bowler D.E., Buyung-Ali L.M., Knight T.M., Pullin A.S. (2010). A systematic review of evidence for the added benefits to health of exposure to natural environments. BMC Public Health.

[9] Hansen M.M., Jones R., Tocchini K. (2017). Shinrin-Yoku (Forest Bathing) and Nature Therapy: A State-of-the-Art Review. Int. J. Environ. Res. Public Health.

[10] Franco L.S., Shanahan D.F., Fuller R.A. (2017). A Review of the Benefits of Nature Experiences: More than Meets the Eye. Int. J. Environ. Res. Public Health.

[11] Song C., Ikei H., Miyazaki Y. (2016). Physiological Effects of Nature Therapy: A Review of the Research in Japan. Int. J. Environ. Res. Public Health.

[12] Ulrich R.S., Simons R.F., Losito B.D., Fiorito E., Miles M.A., Zelson M. (1991). Stress recovery during exposure to natural and urban environments. J. Environ. Psychol..

[13] Kaplan S. (1995). The restorative benefits of nature: Toward an integrative framework. J. Environ. Psychol..

[14] Wilson E.O. (1984). Biophilia.

[15] Ikei H., Song C., Miyazaki Y. (2017). Physiological Effects of Wood on Humans: A Review. J. Wood Sci..

[16] Park S.A., Song C., Choi J.Y., Son K.C., Miyazaki Y. (2016). Foliage plants cause physiological and psychological relaxation as evidenced by measurements of prefrontal cortex activity and profile of mood states. HortScience.

[17] Ikei H., Song C., Igarashi M., Namekawa T., Miyazaki Y. (2014). Physiological and psychological relaxing effects of visual stimulation with foliage plants in high school students. Adv. Hortc. Sci..

[18] Ikei H., Komatsu M., Song C., Himoro E., Miyazaki Y. (2014). The physiological and psychological relaxing effects of viewing rose flowers in office workers. J. Physiol. Anthropol..

[19] Bringslimark T., Hartig T., Patil G.G. (2009). The psychological benefits of indoor plants: A critical review of the experimental literature. J. Environ. Psychol..

[20] Lee J., Park B.J., Tsunetsugu Y., Ohira T., Kagawa T., Miyazaki Y. (2011). Effect of Forest Bathing on Physiological and Psychological Responses in Young Japanese Male Subjects. Public Health.

[21] Park B.J., Tsunetsugu Y., Kasetani T., Kagawa T., Miyazaki Y. (2010). The Physiological Effects of Shinrin-Yoku (Taking in the Forest Atmosphere or Forest Bathing): Evidence from Field Experiments in 24 Forests across Japan. Environ. Health Prev. Med..

[22] Park B.J., Tsunetsugu Y., Kasetani T., Morikawa T., Kagawa T., Miyazaki Y. (2009). Physiological Effects of Forest Recreation in a Young Conifer Forest in Hinokage Town, Japan. Silva Fenn..

[23] Tsunetsugu Y., Lee J., Park B.J., Tyrväinen L., Kagawa T., Miyazaki Y. (2013). Physiological and Psychological Effects of Viewing Urban Forest Landscapes Assessed by Multiple Measurements. Landsc. Urban Plan..

[24] Tsunetsugu Y., Park B.J., Ishii H., Hirano H., Kagawa T., Miyazaki Y. (2007). Physiological Effects of Shinrin-Yoku (Taking in the Atmosphere of the Forest) in an Old-Growth Broadleaf Forest in Yamagata Prefecture, Japan. J. Physiol. Anthropol..

[25] Ulrich R.S. (1984). View through a window may influence recovery from surgery. Science.

[26] Igarashi M., Ikei H., Song C., Miyazaki Y. (2014). Effects of olfactory stimulation with rose and orange oil on prefrontal cortex activity. Complement Ther. Med..

[27] Igarashi M., Song C., Ikei H., Miyazaki Y. (2014). Effects of olfactory stimulation with perilla essential oil on prefrontal cortex activity. J. Altern. Complement. Med..

[28] Ikei H., Song C., Miyazaki Y. (2015). Physiological effect of olfactory stimulation by Hinoki cypress (*Chamaecyparis obtusa*) leaf oil. J. Physiol. Anthropol..

[29] Tsunetsugu Y., Park B.J., Miyazaki Y., Li Q. (2012). Physiological Effects of Visual, Olfactory, Auditory, and Tactile Factors in the Forest Environment. Forest Medicine.

[30] Kimura A., Sugiyama H., Sasaki S., Yatagai M. (2011). Psychological and Physiological Effects in Humans Induced by the Visual and Olfactory Stimulations of an Interior Environment made of Hiba (Thujopsis Dolabrata) Wood. Mokuzai Gakkaishi.

[31] Ikei H., Song C., Miyazaki Y. Physiological effect of contact to Japanese cypress wood with the palm. Proceedings of the 67th Annual Meeting of Japan Wood Research Society.

[32] Lee M.S., Park B.J., Lee J., Park K.T., Ku J.H., Lee J.W., Oh K.O., Miyazaki Y. (2013). Physiological relaxation induced by horticultural activity: Transplanting work using flowering plants. J. Physiol. Anthropol..

[33] Lee M.S., Lee J., Park B.J., Miyazaki Y. (2015). Interaction with Indoor Plants may Reduce Psychological and Physiological Stress by Suppressing Autonomic Nervous System Activity in Young Adults: A Randomized Crossover Study. J. Physiol. Anthropol..

[34] World Health Organization (WHO) Apps. Int/bmi/index.jsp.

[35] Piantadosi S., Steven P. (2005). Crossover designs. Clinical Trials: A Methodologic Perspective.

[36] Task Force of the European Society of Cardiology and the North American Society of Pacing and Electrophysiology (1996). Heart rate variability: Standards of measurement, physiological interpretation, and clinical use. Circulation.

[37] Pagani M., Lombardi F., Guzzetti S., Rimoldi O., Furlan R., Pizzinelli P., Sandrone G., Malfatto G., Dell’Orto S., Piccaluga E. (1986). Power spectral analysis of heart rate and arterial pressure variabilities as a marker of sympatho-vagal interaction in man and conscious dog. Circ. Res..

[38] Kobayashi H., Park B.J., Miyazaki Y. (2012). Normative references of heart rate variability and salivary alpha-amylase in a healthy young male population. J. Physiol. Anthropol..

[39] Watanabe T., Mizuno T., Shikayama T., Miwa M. (2012). Development of a wireless near-infrared tissue oxygen monitor system with high sampling rate. Digital Holography and Three-Dimensional Imaging.

[40] Perry S., Boecker H., Hillman C.H., Scheef L., Strüder H.K. (2012). NIRS for measuring cerebral hemodynamic responses during exercise. Functional Neuroimaging in Exercise and Sport Sciences.

[41] Jöbsis F.F. (1977). Noninvasive, infrared monitoring of cerebral and myocardial oxygen sufficiency and circulatory parameters. Science.

[42] Perrey S. (2008). Non-invasive NIR spectoroscopy of human brain function during exercise. Methods.

[43] Osgood C.E., Suci G.J., Tannenbaum P.H. (1957). The Measurement of Meaning.

[44] McNair D.M., Heuchert J.P., Shilony E. (2003). Profile of Mood States Bibliography 1964–2002.

[45] Osgood C.E. (1952). The nature and measurement of meaning. Psychol. Bull..

[46] Yeun E.J., Shin-Park K.K. (2006). Verification of the profile of mood states-brief: Cross-cultural analysis. J. Clin. Psychol..

[47] Baker F., Denniston M., Zabora J., Polland A., Dudley W.N. (2002). A POMS short form for cancer patients: Psychometric and structural evaluation. Psychooncology.

[48] Taelman J., Vandeput S., Spaepen A., Huffel S. (2009). Influence of mental stress on heart rate and heart rate variability. Proceedings of the 4th European Conference of the International Federation for Medical and Biological Engineering.

[49] Igarashi M., Aga M., Ikei H., Namekawa T., Miyazaki Y. (2015). Physiological and psychological effects on high school students of viewing real and artificial pansies. Int. J. Environ. Res. Public Health.

[50] MacNeilage P.F., Rogers L.J., Vallortigara G. (2009). Origins of the left & right brain. Sci. Am..

[51] Miyazaki Y., Park B.J., Lee J., Osaki M., Braimoh A., Nakagami K. (2011). Nature therapy. Designing Our Future: Local Perspectives on Bioproduction, Ecosystems and Humanity.

[52] Rogers R.L., Meyer J.S., Mortel K.F., Mahurin R.K., Thornby J. (1985). Age-related reductions in cerebral vasomotor reactivity and the law of initial value: A 4-year prospective longitudinal study. J. Cereb. Blood Flow Metab..

[53] Scher H., Furedy J.J. (1985). Individual differences in phasic cardiac reactivity to psychological stress and the law of initial value. Psychophysiology.

[54] Park S.A., Lee K.S., Son K.C. (2011). Determining exercise intensities of gardening tasks as a physical activity using metabolic equivalents in older adults. HortScience.

[55] Park S.A., Lee K.S., Son K.C., Shoemaker C. (2012). Metabolic cost of horticulture activities in older adults. J. Jpn. Soc. Hortc. Sci..

[56] Park S.A., Lee J.Y., Lee K.S., Son K.C. (2014). Metabolic costs of daily activities in community-dwelling older adults. Int. J. Gerontol..

[57] Park S.A., Oh S.R., Lee K.S., Son K.C. (2013). Electromyographic analysis of upper limb and hand muscles during horticultural activity motions. HortTechnology.

[58] Park S.A., Lee A.Y., Kim J.J., Lee K.S., So J.M., Son K.C. (2014). Electromyographic analysis of upper and lower limb muscles during gardening tasks. Korean J. Hortc. Sci. Technol..

[59] Park S.A., Lee A.Y., Son K.C., Lee W.L., Kim D.S. (2016). Gardening intervention for physical and psychological health benefits in elderly women at community centers. HortTechnology.

[60] Park S.A., Shoemaker C., Haub M. (2008). Can older gardeners meet the physical activity recommendation through gardening?. HortTechnology.

[61] Lee A.Y., Park S.A., Kim J.J., So J.M., Son K.C. (2016). Kinematic and kinetic analysis of upper limb motions during horticultural activities. Korean J. Hortc. Sci. Technol..

[62] Park S.A., Lee J.Y., Lee A.Y., Park S.W., Son K.C. (2015). Horticultural therapy program based on the stress immunization training for reducing depression symptom in the patients with stroke. J. Korean Soc. People Plant Environ..

[63] Park S.A., Lee A.Y., Park H.G., Son K.C., Kim D.S., Lee W.L. (2017). Gardening intervention as a low-to moderate-intensity physical activity for improving blood lipid profiles, blood pressure, inflammation, and oxidative stress in women over the age of 70: A pilot study. HortScience.

[64] Bird W. (2004). Natural Fit: Can Green Space and Biodiversity Increase Levels of Physical Activity.

[65] Lekies K.S., Sheavly M.E. (2007). Fostering children’s interests in gardening. Appl. Environ. Educ. Commun..

